# Rectal adenocarcinoma with a yolk sac tumor component: A rare case report and review of the literature

**DOI:** 10.1007/s13691-025-00831-5

**Published:** 2025-12-12

**Authors:** Sato Nishida, Tomohiro Takeda, Tatsuya Shonaka, Shoichiro Mizukami, Masahide Otani, Mizuho Ohara, Chikayoshi Tani, Kimiharu Hasegawa, Yuki Kamikokura, Mishie Tanino, Hideki Yokoo

**Affiliations:** 1 Division of Gastrointestinal Surgery, Department of Surgery, Asahikawa Medical University, 2-1 Midorigaoka Higashi, Asahikawa, 078-8510 Hokkaido Japan; 2 Department of Diagnostic Pathology, Asahikawa Medical University Hospital, Asahikawa, Hokkaido Japan; 3 Division of Hepatobiliary Pancreatic and Transplantation Surgery, Department of Surgery, Asahikawa Medical University, Asahikawa, Hokkaido Japan

**Keywords:** Yolk sac tumor, Extragonadal germ cell tumor, Rectal adenocarcinoma

## Abstract

Yolk sac tumor (YST) occurs primarily in the gonads but rarely outside the gonads. They typically arise in the midline location, and there are only a few reports describing the colon and rectum as the primary sites of YST. Additionally, colorectal cancers containing YST-like components are rare, and in other organs, including the stomach, it is more common to describe such lesions as " yolk sac tumor-like carcinoma”. Here, we report a case of colorectal adenocarcinoma with a YST-like component arising in the rectum. This is the seventh reported case of colorectal YST-like carcinoma. A 66-year-old man presented to our hospital with the chief complaint of anal bleeding. Colonoscopy revealed a 25 × 20 mm tumor located 5 cm above the anal verge. Biopsy results showed moderately differentiated tubular adenocarcinoma, cT3, cN2b, cM0, and cStage IIIC (UICC 8th edition). Subsequently, a laparoscopic abdominoperineal resection was performed. A histopathological examination of the resected specimen revealed findings of adenocarcinoma and YST-like component. Immunostaining for mismatch repair proteins revealed no abnormalities. The tumor was positive for the *KRAS* G12S mutation, and negative for the *BRAF* mutation. The overall diagnosis was adenocarcinoma with a YST-like component, pT3, pN2a, cM0, and pStage IIIB (UICC 8th edition). Six months after the surgery, metastasis was found in the liver, but at the request of the patient and their family, the decision was made not to undergo any aggressive treatment. We describe the clinical and pathological features of colorectal YST-like carcinoma that aid in its accurate diagnosis and provide treatment suggestions.

## Introduction

 Yolk sac tumor (YST) represents 3–5% of ovarian malignancies and occur mainly in children and young women [1]. 24% of YST occur at extragonadal sites [2]. These typically arise in midline locations, such as the mediastinum, retroperitoneum, and brain, and rarely in the colon and rectum [3]. Because there are few reports of YST occurring in the colon and rectum, they are difficult to differentiate. Colorectal cancers containing germ cell components such as neoplastic elements are rare. Histologically, most cases involve a mixture of choriocarcinoma and adenocarcinoma [4]. In this report, we describe an extremely rare case of colorectal adenocarcinoma with a YST-like component and summarize the characteristics and treatment of adenocarcinoma with a YST-like component in comparison to extragonadal colorectal YST.

## Case presentation

A 66-year-old man presented to our hospital with the chief complaint of anal bleeding. Symptoms developed 1 month before presentation, marked by anal bleeding. He had a history of frontotemporal dementia and ADL equivalent to performance status (PS) 2. His older brother had digestive system cancer, but the details were unknown. The patient’s height and weight were 1.68 m and 72.0 kg, respectively, and his body mass index was 25.5 kg/m². A rectal examination revealed a mass on the anterior rectal wall. Blood biochemical tests were normal, while tumor marker tests revealed carbohydrate antigen 19 − 9 11 U/mL, carcinoembryonic antigen (CEA) 7.7 U/mL, and an elevated CEA level.

**Fig. 1:**
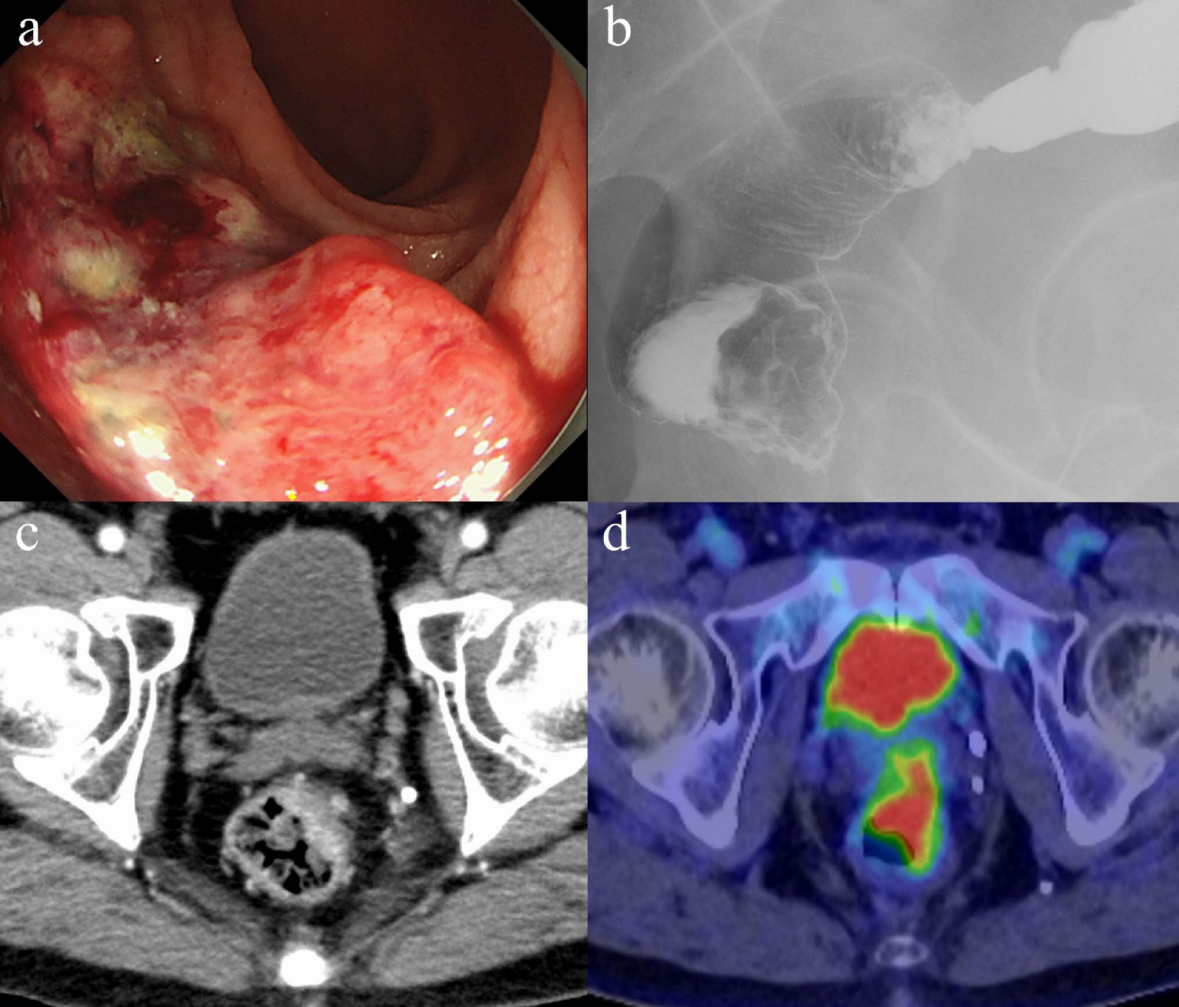
Results of the examination before the surgical treatment. a: Colonoscopy revealed a 25 × 20 mm tumor was located 5 cm above the anal verge on the anterior wall of the lower rectum. b: Gastrografin enema showing deformity in the anterior wall of the lower rectum. c: Contrast-enhanced CT tomography showing irregular wall thickening from the anterior to the left wall of the lower rectum (approximately half the circumference). d: Positron emission tomography-CT showed abnormal accumulation in the left wall of the anterior wall of the lower rectum

Colonoscopy revealed that the tumor, measuring 25 × 20 mm, was located 5 cm above the anal verge on the anterior wall of the lower rectum. (Fig. 1a). A biopsy revealed moderately differentiated tubular adenocarcinoma, and genetic testing revealed a *KRAS* G12S mutation. A gastrografin enema showed wall deformity in the anterior wall of the lower rectum (Fig. 1b). Computed tomography (CT) showed irregular wall thickening from the anterior to the left wall of the lower rectum (approximately half the circumference) and multiple enlarged lymph nodes from the superior rectal artery and left internal iliac region. (Fig. 1c). Positron emission tomography (PET)-CT showed abnormal accumulation (SUV_max_ 13.0) in the left wall from the anterior wall of the lower rectum, abnormal accumulation (SUV_max_ 8.4) in the enlarged lymph nodes around the perirectal to superior rectal artery, with no significant increase in accumulation in the lymph nodes in the left internal iliac artery region. No evidence of distant metastasis was observed (Fig. 1 d).

Based on these findings, the pretreatment diagnosis was lower rectal cancer cT3, cN2b, cM0, and cStage IIIC (UICC 8th edition) [5]. Laparoscopic abdominoperineal resection with left lateral lymph node dissection was performed. The intraoperative findings revealed no obvious peritoneal dissemination or liver metastasis.

**Fig. 2:**
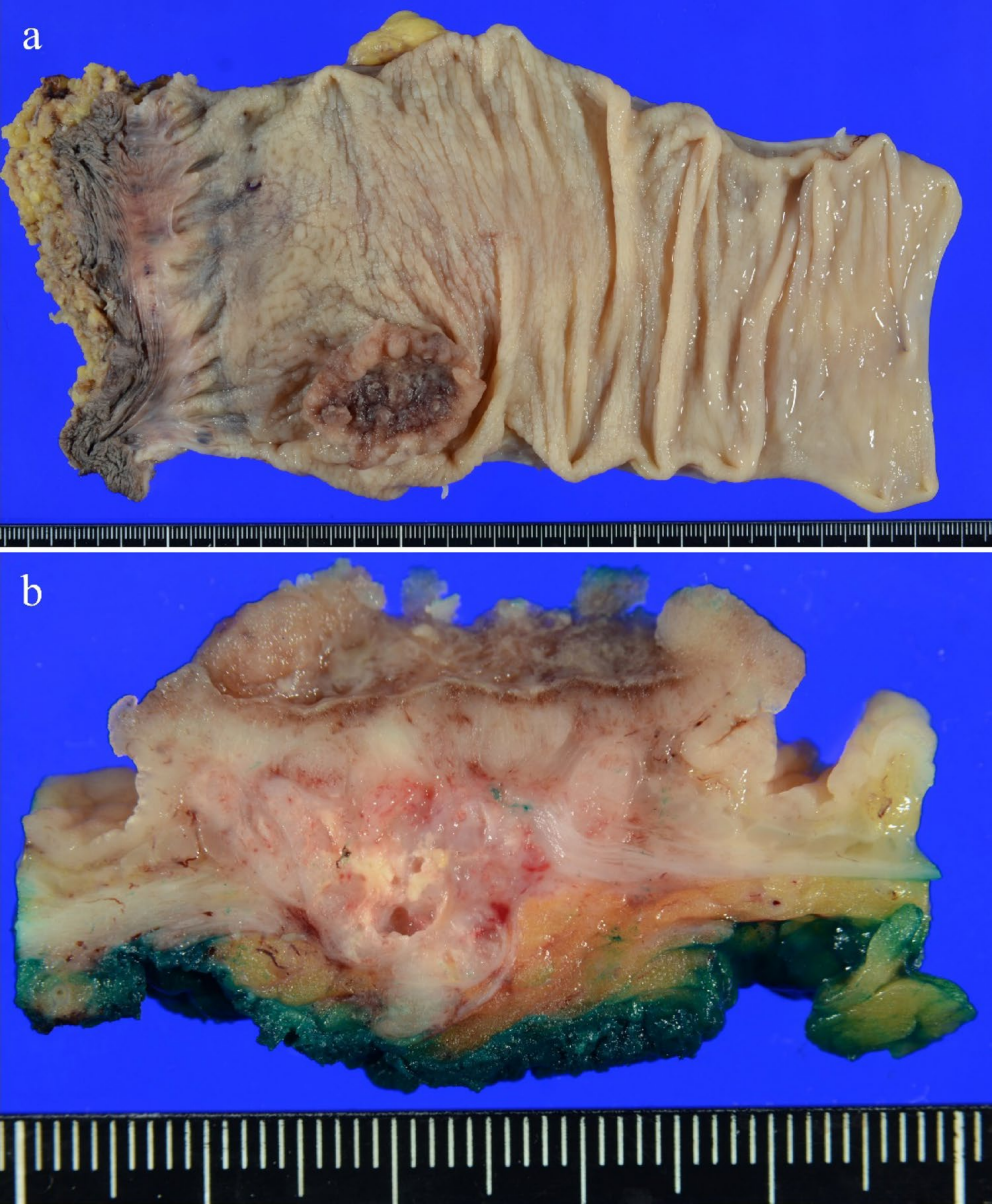
Surgical specimen. a: The resected specimen showed a 39 × 34 mm tumor with an ulcer in the lower rectum 48 mm from the anal margin. b: The cut surface of the tumor

**Fig. 3:**
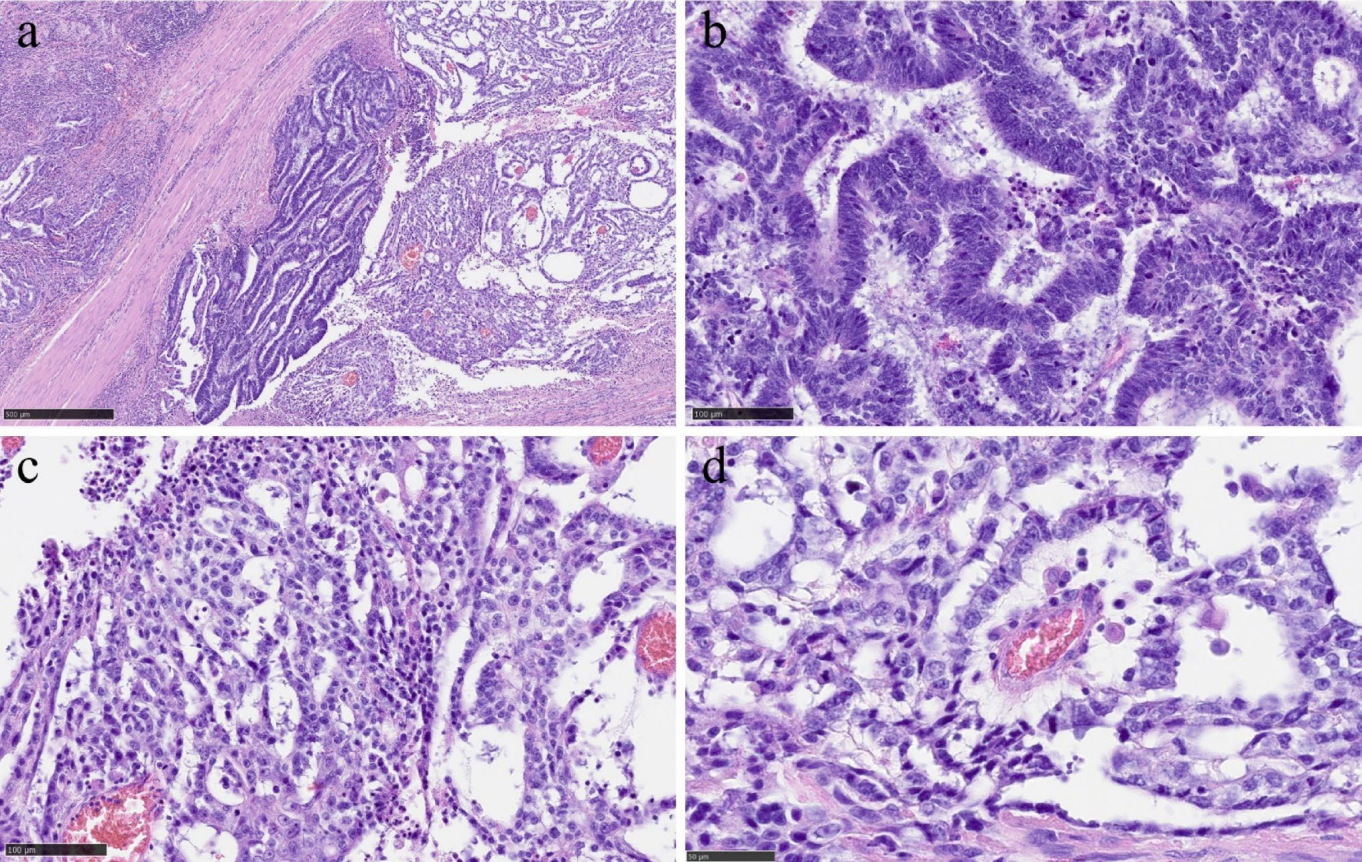
Pathohistological findings. a: Adenocarcinoma and yolk sac tumor component adjacent to each other (HE staining, 50×) b: Histological findings of adenocarcinoma include infiltration of atypical columnar cells with enlarged nuclei and coarse chromatin, forming fused and cribriform glandular ducts, as well as irregular glandular ducts. (HE staining, 200×). c Histological findings of the yolk sac tumor: atypical cells with rounded nuclei, hyperchromasia, and nucleoli were observed, and atypical cells with light to pale eosinophilic cytoplasm proliferated in the reticulate, tubular, and papillary structures. (HE staining, 200×). d: Schiller Duval Body. (HE staining, 400×)

**Fig. 4:**
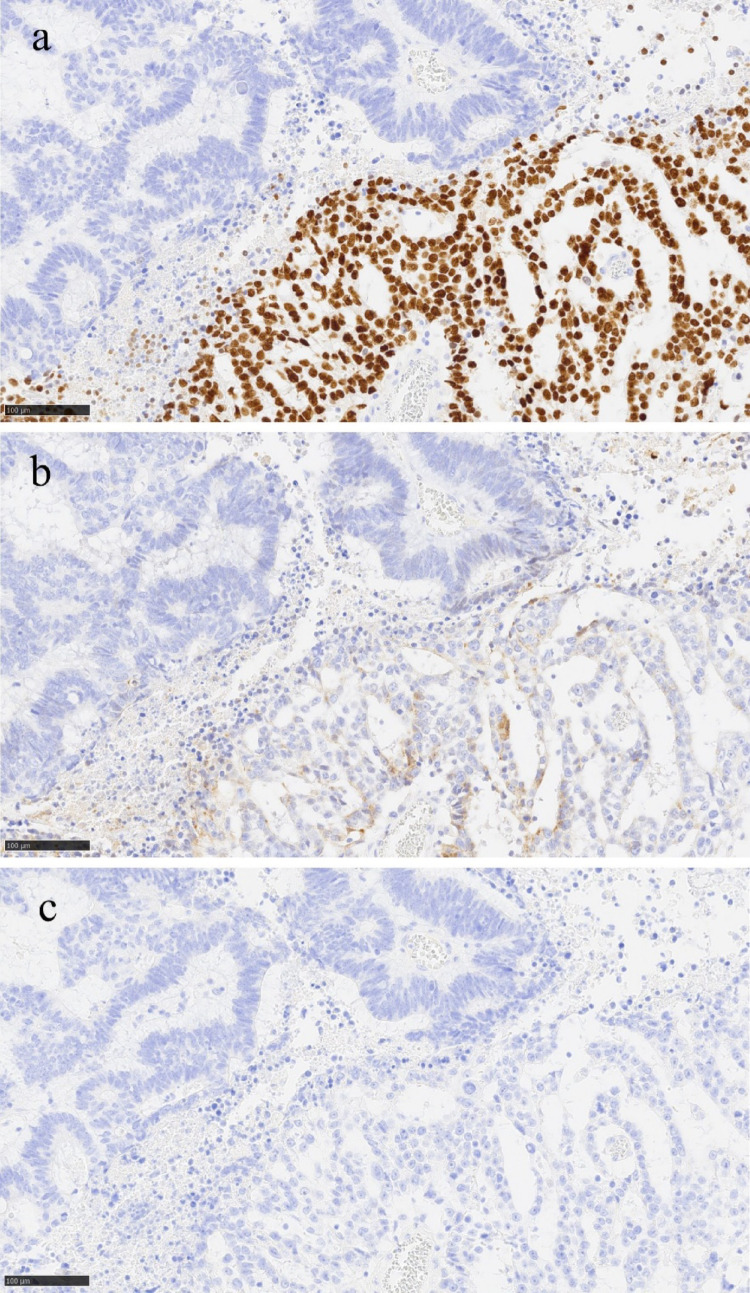
Immunostaining results. a: Positive for SALL4. (200×) b: Partially positive for Glypican3. (200×) c: Negative for AFP. (200×)

The resected specimen showed a 39 × 34 mm tumor with an ulcer in the lower rectum, 48 mm from the anal margin (Fig. 2a-b). Histopathological findings showed infiltration of atypical columnar cells with enlarged nuclei and coarse chromatin, forming fused and cribriform glandular ducts and irregular glandular ducts. Microscopic examination revealed small nests and single-cell infiltration with minimal fibrous stroma, villous structures, tumor cells floating within mucin pools, and tumor cells floating in the mucus lake. The tumor was diagnosed as moderately differentiated tubular adenocarcinoma (tub2 > > tub1 >muc >pap >por2) (Fig. 3a-b). In approximately 5% of the tumors, atypical cells with rounded nuclei, hyperchromasia, and nucleoli were observed. Atypical cells with light to pale eosinophilic cytoplasm proliferated in the reticulate, tubular, and papillary structures (Fig. 3c). Schiller-Duval body-like structures were also observed (Fig. 3 d). Immunostaining of this area was diffusely positive for SALL4 (Spalt-like transcription factor 4) (Fig. 4a), partially positive for Glypican3 (Fig. 4b), and negative for alpha-fetoprotein (AFP) (Fig. 4c). Based on these results, this area was confirmed to be a YST-like component. The overall diagnosis was adenocarcinoma (tub2 > > tub1 >muc >pap >por2) with a YST-like component, pT3, pN2a, cM0, and pStage IIIB (UICC 8th edition) [5]. Immunostaining for mismatch repair proteins revealed no abnormalities. The tumor was positive for the *KRAS* G12S mutation and negative for the *BRAF* mutation. The patient had a good postoperative course and was discharged on the 23rd postoperative day.

Because of cognitive decline due to frontotemporal dementia and a PS score of 2, the patient did not receive postoperative adjuvant chemotherapy and was observed for follow-up. The postoperative serum AFP level was 1.0 ng/mL, which was within the standard range. Six months after the surgery, metastasis was found in the liver; however, at the request of the patient and their family, the decision was made not to undergo any aggressive treatment.

## Discussion

Although AFP production is rare in Gastric cancer, it has reported, and cases that exhibit pathological findings similar to YST are sometimes referred to as YST-like carcinomas. Most gastric YST-like carcinomas are thought to be associated with an adenocarcinoma component [6]. In this report, we define cases in which colorectal adenocarcinoma and YST-like component coexist as colorectal YST-like carcinoma, and consider them in comparison with extragonadal pure colorectal YST.

The results of a literature search for YST in the colon and rectum are summarized in Table 1. Only 16 cases of YST in the colon and rectum have been reported, including the present case. Colorectal adenocarcinoma with YST-like component (colorectal YST-like carcinoma) accounted for 7 cases, including the present case. The median age at presentation was 28 years (range, 22–54 years) for pure colorectal YST and 49 years (range, 18–66 years) for colorectal YST-like carcinoma. In colorectal pure YST, 86% of cases occur in females, whereas colorectal YST-like carcinomas show no sex predilection. The most common site was the rectum, followed by the sigmoid colon, with similar trends in both cases. Thirteen patients (81%) underwent surgical resection. At the initial diagnosis, the disease stages (UICC 8th edition) [5] were as follows: Stage I (*n* = 1), Stage IIA (*n* = 1), Stage IIIA (*n* = 1), Stage IIIB (*n* = 1), and Stage IV (*n* = 11). Of the cases with distant metastasis, 90% tended to occur in the liver, and all cases of colorectal YST-like carcinoma had liver metastasis. This case also showed metastasis to the liver, thus suggesting the importance of carefully following up such cases for liver metastasis.

**Table 1:** Reports of yolk sac tumor of the colon and rectum

No	Author	Year	Age	Sex	Location	pStage*	Metastasis	Non-YST histology	AFP (ng/mL)	AFP IHC	SALL4	Glipican3	Operation	Chemotherapy	NAC	Prognosis	Observation period
1	Lankerani MR [13]	1982	42	F	S	**	-	choriocarcinoma + teratoma		+			+	DOX + CB + MTX	-	Dead	96 months
2	Yu YY [9]	1992	54	M	R	Ⅳ	liver	adenocarcinoma	5126	+			+	-	-	Dead	5 days
3	Ostör AG [11]	1993	28	F	R	Ⅳ	liver	adenocarcinoma + choriocarcinoma	300	+			+	EMACO	-	Dead	3 months
4	Miller KD [10]	2000	23	F	R	Ⅰ	-		2251 × 10⁵	+			+	-	BEP	Alive	42 months
5	Petricek CM [4]	2001	29	M	T	Ⅳ	liver	adenocarcinoma + choriocarcinoma	745,732	+			-	-	-	Dead	6 days
6	Tseng MJ [8]	2007	27	F	R	ⅡA	-		4830****	-			+	PE	-	Alive	12 months
7	Kawahara M [7]	2009	62	M	A	Ⅳ	liver	adenocarcinoma + choriocarcinoma	553	+			+	5FU	-	Dead	4 months
8	Rudaitis V [12]	2016	22	F	R	Ⅳ	liver, peritoneum, greater omentum			+			+	-	BEP	Alive	24 months
9	Bansal A [16]	2016	28	F	S	Ⅳ	liver		210	+			+	BEP	-	Alive	96 months
10	Huang Q [2]	2018	29	F	R	Ⅳ	liver		1210	+	+	+	-	BEP	-	Alive	27 months
11	Vishnoi V [15]	2018	24	M	C	ⅢA	-		2145	+		+	+	-	-	Alive	3 days
12	Zaki MMAF [18]	2020	18	F	***	Ⅳ	liver	adenocarcinoma	1200								
13	Short R [3]	2022	54	F	C	Ⅳ	retroperitoneum			+			+	BEP	-	Alive	6 months
14	Otani T [14]	2022	49	F	S	Ⅳ	ureter, lung, liver, bone, peritoneum	adenocarcinoma	42	+	+	+	+	TC + Bev + DOX, FOLFOXIRI + Bev, FTD/TPI+ Bev	-	Dead	14 months
15	Muddasetty R [17]	2024	34	F	S	Ⅳ	liver		8930****	-			+	BEP	FOLFOX	Alive	3 months
16	Our case	2025	66	M	R	ⅢB	liver	adenocarcinoma	1****	-	+	+	+	-	-	Alive	7 months

* UICC 8th, postoperative diagnosis, diagnosis at the time of biopsy if no surgery is performed

** Arising from the serosa. No other information. Difficult to stage

*** Unable to confirm

**** Postoperative serum AFP level

Abbreviations: YST, yolk sac tumor; AFP, a-fetoprotein; SALL4, Sal-like protein 4; NAC, neoadjuvant chemotherapy: S, sigmoid colon; R, rectum; T, transverse colon; A, ascending colon; C, cecum; DOX + CB + MTX, doxorubicin + chlorambucil + methotrexate; EMACO, etoposide + methotrexate + actinomycin-D + cyclophosphamide + vincristine; PE, cisplatin + etoposide; 5FU, 5-Fluorouracil; BEP, bleomycin + etoposide + cisplatin; TC, docetaxel + cyclophosphamide; Bev, bevacizumab; DOX, doxorubicin; FOLFOXIRI, fluorouracil + oxaliplatin + leucovorin + irinotecan; FTD/TPI, trifluridine/tipiracil

The mechanism underlying the coexistence of adenocarcinoma and germ cell tumors is not clear, but there are two hypotheses [7]. The first is a collision tumor in which two independent tumors coexist by chance. Specifically, they are thought to arise from misplaced germ cells, as they migrate during development. This also explains why germ cell tumors are more common in the midline. The second is dedifferentiation, in which the genetic instability of the tumor cells alters the structure and regulation of the genome, leading to dedifferentiation into germline cells, from which they arise. This explains its occurrence outside the midline. In the present case, the tumor was not located at the midline, and histologically, it was adjacent to an adenocarcinoma, suggesting that it was caused by dedifferentiation.

Although the serum AFP level has been reported to be positive in all cases of YST of the ovary [1], in our case AFP immunostaining and the serum AFP level was negative. With the exception of our case, all reported cases were serum AFP positive, while in 2 of those cases, immunostaining was negative. AFP immunostaining is used to confirm AFP production in tumor tissues, but not all tumors with an elevated serum AFP level show positive tissue staining due to a heterogeneous intratumoral AFP expression [19]. In hepatocellular carcinoma, which is known to have a high frequency of AFP-producing cancers, only about 40% of the serum AFP positives were also positive for immunostaining [19]. Therefore, it is important to measure the serum AFP level or perform histological examinations even if immunostaining is negative. The preoperative serum AFP level may therefore have been positive in this case as well. Colon and rectal adenocarcinoma with enteroblastic differentiation (CAED) is the most common type of AFP-producing colorectal tumor [14, 20]. CAED is a rare subtype of colonic adenocarcinoma characterized by increased AFP production and expression of at least one enteroblastic marker, including AFP, glypican 3, or SALL4. It has been characterized histologically as having a primitive intestine-like structure composed of cuboidal or columnar cells with a clear cytoplasm. CAED rapidly progresses and metastasizes to the liver, and is associated with a poor prognosis. Although CAED and colorectal YST-like carcinoma share clinical features, they can be distinguished based on pathological findings [20].

YST pathology is characterized by multiple histological patterns within the same tumor [21]. The most common types are microcystic, reticular, glandular, and solid patterns, whereas papillary and hepatoid patterns are rarer [21]. Most cases, including the present case, were proliferating, forming microcystic and reticular patterns. Although papillary patterns are relatively rare, they were found in 5 cases of pure colorectal YST (56%) and 4 cases of colorectal YST-like carcinoma (57%), including our own. It is unclear whether this was due to differences in the site of origin or bias due to the small number of cases. Schiller-Duval bodies are also characteristic, but are present in only 20% of YST [21]. Schiller-Duval bodies were found in 3 cases of pure colorectal YST (33%) and 4 cases of colorectal YST-like carcinoma (57%). Immunostaining is also important; common immunostaining for YST includes AFP, Glypican 3, and SALL4, with sensitivities of 83%, 69%, and 100%, respectively, with SALL4 being the most sensitive [21]. The findings in this case were consistent with the aforementioned histopathological features and immunostaining results, leading to a diagnosis of YST-like component.

While no cases of colorectal pure YST, in which patients died within 6 months of surgery, have been reported, the median survival time for colorectal YST-like carcinoma was 6 months, excluding cases of early postoperative death. These findings suggest that colorectal YST-like carcinoma has a poor prognosis, similar to that of CAED.

Contemporary treatment protocols for YST include four cycles of bleomycin, etoposide, and cisplatin (BEP) or ifosfamide, etoposide, and cisplatin (VIP), followed by surgical resection of the residual masses [22]. This treatment is often used for extragonadal YST [23]. Kathy et al. diagnosed a primary YST of the rectum using endoscopic biopsy and reported that the patient underwent surgical resection after BEP therapy for 3.5 years without recurrence [10]. When extragonadal germ cell tumors are treated with preoperative chemotherapy followed by surgical resection, 56% of patients have no residual tumor in the resected specimen, indicating that chemotherapy is highly effective [24]. As demonstrated by this case and supported by existing literature, YST-directed chemotherapy significantly improves the prognosis of extragonadal YST. On the other hand, although adjuvant chemotherapies including cisplatin, vinblastine, bleomycin, and etoposide according to germ cell tumor treatment were performed for advanced gastric YST-like carcinoma, they have not been shown improve long-term survival [6]. Although there is a possibility that it may be less effective in also colorectal YST-like carcinoma, no cases of colorectal YST-like carcinoma have been treated with BEP or VIP, it is unclear whether treatment for colorectal YST-like carcinoma should prioritize the adenocarcinoma or the YST-like component. It is not easy to diagnose colorectal YST-like carcinoma based on preoperative examination alone, since the findings of adenocarcinoma often dominate the findings of YST, and it is important to perform an appropriate specimen evaluation with consideration of postoperative treatment options [17].

YST-like component occurring in the colon and rectum with adenocarcinoma is rare, and the characteristic clinical manifestations and diagnostic clues are not well understood. To avoid missing colorectal YST-like carcinoma, AFP could be measured as a screening measure but given its relative infrequency and the fact that some YST is AFP-negative, routine measurement for suspected colorectal cancer is of little significance. However, if a preoperative biopsy or postoperative histological analysis shows an image that is different from that of a normal colorectal adenocarcinoma, the possibility of colorectal YST or colorectal YST-like carcinoma should be considered, and the measurement of serum AFP or immunostaining may be useful.

Here, we describe a case of rectal adenocarcinoma with　a YST-like component. The preoperative diagnosis of colorectal YST-like carcinoma remains challenging; however, accurate postoperative diagnosis is important to provide optimal treatment. More studies are needed to identify rare cases of colorectal YST-like carcinoma and to establish a standard of care.

## Data Availability

No datasets were generated or analyzed during the current study.

## Abbreviations

YST: Yolk sac tumor
PS: Performance Status
CEA: carcinoembryonic antigen
CT: Computed tomography
PET: Positron emission tomography
SALL4: Spalt-like transcription factor 4
AFP: Alpha-fetoprotein
BEP: bleomycin, etoposide, and cisplatin
VIP: ifosfamide, etoposide, and cisplatin
